# Barriers to help-seeking in medical students with anxiety at the University of South Carolina School of Medicine Greenville

**DOI:** 10.1186/s12909-023-04460-5

**Published:** 2023-06-21

**Authors:** Helen Kaiser, Tori Grice, Brittany Walker, Jacob Kaiser

**Affiliations:** 1 University of South Carolina School of Medicine Greenville, Greenville, SC 29605 USA; 2 Prisma Health Upstate, 701 Grove Road Balcony Suite 5, Greenville, SC 29605 USA; 3 Prisma Center for Psychiatry, 109 Physicians Dr. Ste B, Greer, SC 29650 USA

**Keywords:** Medical school, Anxiety, Stigma, Self-Stigma, Mental health

## Abstract

Anxiety levels in medical students have been reported as higher than the aged-matched general population, yet medical students are less likely to seek care for mental health issues. Medical students carry high levels of self-stigma about their own mental health and fear the negative consequences of seeking care. The purpose of this study was to examine the student population at the University of South Carolina School of Medicine Greenville (UofSC SOMG) for anxiety levels and determine the self-stigma attitudes this population carries. UofSC SOMG students were surveyed using the GAD7, questions about mental health stigma, and open-ended questions on barriers to mental health care in medical students. Anxiety levels were compared to student responses. 31% of students reported moderate-severe anxiety levels. Stigma was the most frequently listed barrier to care, however, students with moderate-severe anxiety were more likely to report cost as a barrier to care than students with minimal anxiety levels. Despite free and accessible mental health care, medical students at UofSC SOMG still have anxiety at rates higher than the general population. Future work should help to provide interventions to the barriers of care, so medical students can better utilize mental health care resources.

**Supplementary Information:**

The online version contains supplementary material available at 10.1186/s12909-023-04460-5.

## Introduction

Anxiety disorders are debilitating conditions that can exhibit psychosomatic symptoms such as fatigue, palpitations, and nausea, as well as impairing goal-directed attention, concentration, working memory, and perceptual-motor function [1–5]. In the US, 4.3% of adults aged 18–29 experience moderate anxiety symptoms, and 3.1% experience severe anxiety symptoms [6]. However, a meta-analysis of anxiety symptoms in medical students globally found that 33% of medical students experience moderate-severe anxiety symptoms [5]. Factors contributing to higher levels of anxiety include high levels of pressure to perform academically, financial concerns, exposure to death and dying, and lifestyle stressors like sleep deprivation, and lack of exercise due to time constraints [4, 7–9]. Furthermore, medical schools may self-select for personalities more prone to developing anxiety disorders [10].

As awareness of mental illness in medical students has increased, so have initiatives to provide mental health resources to students [11, 12]. Many schools have increased education to students about lifestyle wellness, and how to decrease anxiety. In addition, many schools now provide free counseling services to students. At the University of South Carolina School of Medicine-Greenville (USC SOMG) students have access to the Prisma Health Employee Assistance Program (EAP), which provides free counseling sessions to students 24/7 via phone, or in person, and is not directly associated with the university. Students may also access a free psychiatric appointment with an out-of-system psychiatrist. These resources are given to students during orientation and listed on the school website. The phone number to the EAP, as well as the suicide hotline is posted in the hallways at the school, and in the weekly student newsletters.

Despite increased education and resources, many medical students are less likely to seek help for mental health symptoms due to the stigma associated with mental health [5]. Many physicians report that if they were experiencing mental health symptoms they wouldn’t want others to know [2, 3, 13–16]. In 2003 a study at the University of Manchester found 50% of students feared disclosing their mental illness would be risky [15].

We sought to understand if the initiatives in place at UofSC SOMG may have improved the levels of self-stigma and help-seeking behaviors. Further, we sought to use the Generalized Anxiety Disorder 7 question questionnaire (GAD7) to screen the students for generalized anxiety disorder, and by separate survey determine if levels of self-stigma were associated with levels of anxiety. We hypothesized that our students would have lower levels of anxiety than the previously studied populations and that those with higher levels of anxiety would be less likely to seek help due to self-stigma. Finally, an open-ended question was used to elucidate perceptions of barriers to care for mental health.

## Materials and methods

The USofC SOMG is an LCME accredited medical school with two years of pre-clerkship curriculum (M1 and M2), and 2 years of clerkship curriculum (M3 and M4).

A cross-sectional survey was put in the student newsletter during the fall 2021 semester at the UofSC SOMG. 429 students were enrolled at the time the study was conducted. The respective class sizes were: 108-M1, 118-M2, 101-M3, and 102-M4.

The study was approved as exempt by the Health Sciences South Carolina institutional review board. Students were presented with a description of the study, potential harm (minimal), and their right to decline participation at any moment during the survey. Students were able to skip a question or a section if they wanted. Students received no compensation and the only identifying questions were class year, and gender identity. Any further demographic questions were excluded to protect student’s privacy.

Survey design consisted of Likert-style questions, the GAD-7, and an open-ended response question, “If you were suffering from a mental health disorder, what would be the largest barrier to seeking mental health care for yourself?”. Significant survey items from Schwenk et al. were used to begin survey design, then a group of students reviewed the items and adjusted language for relevance at UoSC SOMG [16]. The survey items were 22 statements regarding mental health and stigma, with Likert-style responses. Items were framed with some positive and some negative to check for consistency. The GAD7 questionnaire was not changed. The full survey including the GAD7 questionnaire is available in Supplemental Fig. 1.

Qualtrics was used to create and administer the survey (Qualtrics, Provo, UT). All data was kept on a password-protected computer in a locked office. The survey took 10–15 min to complete and could be accessed on a smartphone through a QR code, or on a computer through a link sent to students. The first introduction to the survey included the language “If you begin the survey, you are free to stop taking the survey at any time.” To allow students to opt-out. Students were informed of the study in their weekly student newsletter. M1 students were also informed of the survey in-person after a class session. They were informed verbally that survey participation would be voluntary, and not affect their performance or grades in any way.

Due to the sensitive nature of some questions, existing mental health resources were provided to students in the newsletter next to the study link. The survey was anonymous so there was no mechanism for follow-up. Existing medical school resources include access to 24/7 counseling services from an entity separate from the school, as well as access to free psychiatry appointments from a psychiatrist not associated with the school. The suicide hotline number was also provided. All mental health care resources are provided to students at orientation at the beginning of each year and are shown on screens in the hallways of the medical school buildings.

The open ended-question responses were categorized into 4 categories: Cost, Stigma, Time, and difficulty. These categories were chosen based on the responses. The difficulty category consisted of those who perceived the largest barrier of care was navigating the system or accessing mental health care. Each of the authors categorized the open-ended questions blinded from each other, and then responses were compared using a Cohen’s kappa. Some students listed more than one answer, and each answer was given a category.

The Generalized anxiety disorder scale -7 (GAD7) is a seven-question screening tool for generalized anxiety disorder. The GAD7 asks if people have suffered symptoms that are diagnostic criterion from the DSM-5 [17] for GAD in the last two weeks. The answer choices are described as ‘not at all’, ‘on some days’, ‘on more than half of the days’ and ‘almost every day’. The GAD7 scores were interpreted as 0–4 for none to minimal anxiety, 5–9 for mild anxiety, 10–14 for moderate anxiety, and 15–21 for severe anxiety. Anxiety scores were collapsed into two categories: none-mild and moderate-severe, as a score of moderate or higher has 99% sensitivity and 82% specificity for a diagnosis of Generalized Anxiety Disorder [13]. The GAD7 has been validated over large patient populations and internal consistency has been reported over (α = 0.8) from multiple studies and populations [18–20].

Stigma questions were compared to class year, gender, and anxiety levels using χ^2^ analyses and Fisher exact tests as appropriate. Class year was coded as a dichotomous variable to compare results for students in preclinical vs clinical education. Multiple comparisons with Bonferroni post hoc tests were used to limit Type I errors. With the experimental error rate set at 0.05, the individual error rate was reduced to 0.002 (0.05 divided by 22 stigma items). Quantitative analyses were performed using SPSS 17.0 statistical software package (SPSS Inc, Chicago, Illinois).

## Results

### Demographics

The overall response rate for the survey was 163 of 429 matriculated students (38%). Not all sections were completed by all respondents. The open-ended questions were answered by 98 respondents. The GAD7 was answered by 117 respondents. The stigma perception questions were answered by 119 respondents. Demographic data was answered by 154 respondents.

The response rate was higher in the preclinical students than the clinical students (42%- M1, 29%-M2, and 14%-M3, 16%-M4 of 154 respondents).

The overall response rate was 60% female, 33% male, 2% third gender or non-binary, and 5% not listed. This response rate was somewhat representative of the student population at UofSC SOMG, as 57% of the student body is reported female, and 43% of the student body is reported male. Of note, non-binary or other gender option demographics are not available for the student body.

### GAD7 and stigma perceptions

**Fig. 1:**
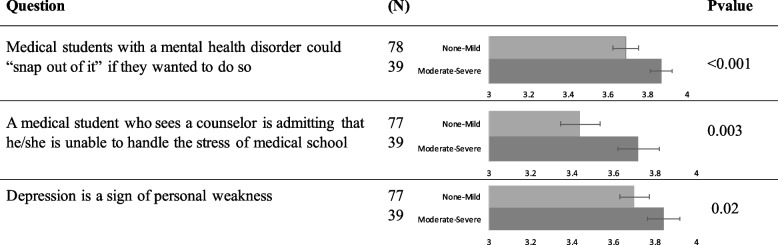
Stigma questions with differences in opinions by anxiety score. Results are presented as mean, with 3 corresponding to “disagree”, and 4 corresponding to “strongly disagree”. One hundred seventeen students with GAD7 scores answered the first question, whereas 116 students with GAD7 scores answered the remaining two questions

One hundred seventeen students completed the GAD7. A total of 39 students (33%; 95% confidence interval (CI), 9%-23%) scored in the moderate-severe anxiety range on the GAD7. Stigma perceptions varied by score. Of the 21 stigma items assessed, 3 items showed significant differences by GAD7 score after Bonferroni adjustment. Students with higher anxiety scores strongly disagreed more than those with none-mild anxiety “Medical students with a mental health disorder could ‘snap out of it’ if they wanted to do so.” (Mean 3.87 vs. 3.69, *p* < 0.001), that “A medical student who sees a counselor is admitting that he/she is unable to handle the stress of medical school” (Mean 3.72 vs. 3.44, *P* = 0.003) and “Depression is a sign of personal weakness” (Mean 3.85 vs. 3.70, *P* = 0.02) (Fig. 1). Stigma perceptions did not differ by gender or class year.

### Open-ended stigma responses

**Fig. 2:**
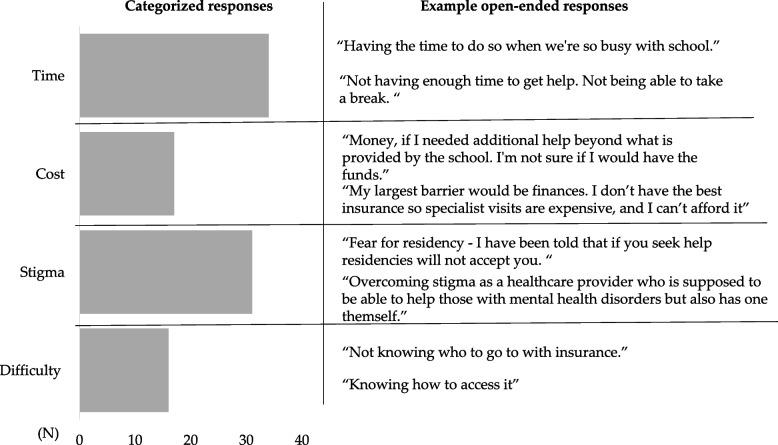
Example answers to open-ended question “If you were suffering from a mental health disorder, what would be the largest barrier to seeking mental health care for yourself” shown in the categories they were assigned to. 34 responses were in Time. 17 responses were in Cost. 31 Responses were in Stigma. 16 responses were in Difficulty (Cohen’s k = 0.644-.930)

**Fig. 3:**
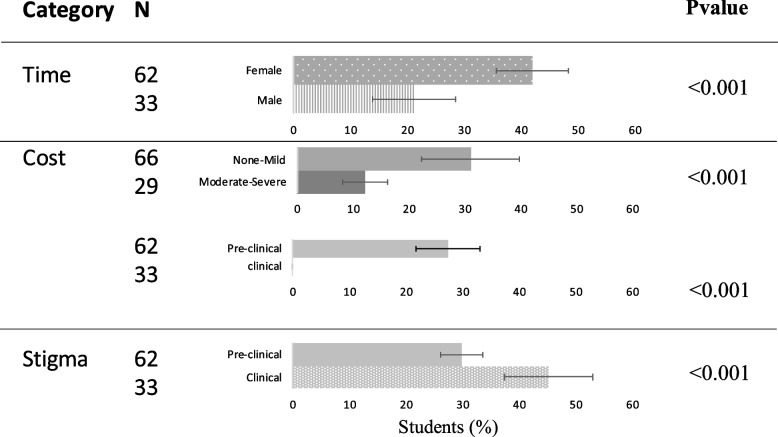
Variables with significant differences are based on open-ended categories. Shown as the percentage of answers in each category. (N = Total number of students responding to both survey section listed and open-ended question Students who filled out both demographic and open-ended sections = 95, Students who filled out GAD7 and open-ended questions = 95.)

Students were asked the open-ended question, “If you were suffering from a mental health disorder, what would be the largest barrier to seeking mental health care for yourself?” Answers were categorized into categories, “Time”, “Cost”, “Stigma”, and “Difficulty”. 98 students responded to the open-ended questions. Agreement between authors regarding categorizing open-ended answers was substantial to perfect (Cohen’s k = 0.644-0.930). Examples of open-ended answers are listed in Fig. 2. Students listed time (34) cost (17), stigma (31) and difficulty (16) Categorical answers were then compared to anxiety levels, class ranking and reported gender and are shown in Fig. 3. Students who did not fill out both demographic and open-ended questions or GAD7 and open-ended questions were excluded. Students with moderate-severe anxiety reported cost as a barrier more often than those with none-mild anxiety (*P* =  < 0.001). Students in Preclinical years answered Cost (*P* =  < 0.001) more than students in clinical years, however students in clinical years answered Stigma (*P* =  < 0.001) more than students in preclinical years. Female students answered time (*P* =  < 0.001) more than male students.

## Discussion

Chronic, unrelenting stress can expedite psychological distress in the form of anxiety disorders and major depressive disorder [21]. Medical school produces a number of stressors for students. Medical students are often living away from their families and support systems [22], They are under high levels of financial strain [23], They are often not in control of their schedules and often medical students were the top of their class in college, but struggle to keep up with the coursework in medical school [24]. High workloads can keep medical students from engaging in healthy lifestyle factors that reduce stress, like exercise, healthy diets, sleep quality, and relationships [5, 13, 22, 24–26]. Medical schools have made significant efforts to support students and provide stress management and relief. Many schools like UofSC SOMG offer in-house success coaches, and non-affiliated psychotherapy. Despite the availability of mental health support, many medical students do not use the resources provided [5]. Several studies have identified that medical professionals and students will not seek therapy due to self-stigma [2, 3, 13–16].

Mental health stigma and self-stigma (internalized public stigma) are higher in the medical community than the general population [5]. Physicians have higher rates of mental health stigma, and many report that they will not seek help for mental health [2, 3, 13–16]. Medical students report fear that disclosing a mental health problem will decrease their chances of getting a residency position [27]. Medical board licensing exams in many states ask directly if a candidate has been treated for a mental health disorder [13].

In this study, we surveyed students at the UofSC SOMG to screen for generalized anxiety disorder using the GAD7. 33% of UofSC SOMG students surveyed scored in the moderate-severe anxiety range on the GAD7. This is a similar rate to previous studies that have screened medical students for anxiety (31%), but much higher than aged-matched peers from the general population in 2021 (15.3%) [28]. To protect medical students’ mental health, it is important to pursue further interventions.

We surveyed students about their feelings of self-stigma and compared their anxiety levels to responses. Students self-reporting higher levels of anxiety also reported stronger disagreement with self-stigma questions. This suggests that students with higher levels of anxiety may carry less self-stigma than those with lower anxiety levels. Stigma perceptions did not differ by gender or class year.

To elucidate student perception of barriers to mental health care in medical school, students were asked the open-ended question, “If you were suffering from a mental health disorder, what would be the largest barrier to seeking mental health care for yourself?” Answers were then categorized into “Time”, “Cost”, “Stigma”, and “Difficulty”. Students listed time (47%), cost (27%), stigma (60%), and difficulty (21%) (some students listed more than one answer, and both answers were categorized separately.) Students with moderate-severe anxiety reported cost as a barrier more often than those with none-mild anxiety. It is possible that students with lower financial support may be more likely to have higher levels of anxiety in medical school. Financial worries are one of the largest stressors [23]. It also may be that students with higher levels of anxiety are more likely to worry about finances, noting that the therapy and psychiatry sessions provided by the school are free. Furthermore, students in preclinical years also answered cost more than students in clinical years. We suggest that as students get further along in their education, the “light at the end of the tunnel” might relieve some anxiety towards paying off high levels of medical school debt, and students may feel less burdened by their debts. Further work should be done to elucidate financial stress in medical students, and how it could be relieved in the earlier years. Interestingly, students in clinical years were more likely to answer “stigma” than students in preclinical years. This may be due to impending residency applications, or due to experiences, students encounter working in a clinical setting around others that carry mental health stigma.

There were several limitations of this study; the 2020 COVID-19 pandemic was reported to increase anxiety levels at different rates in different populations. Due to this, the impact of increased stressors was not able to be controlled. Furthermore, the high level of stigma responses in the open-ended questions may be due to the order of questions asked, with the open-ended questions coming after the stigma survey. And finally, this survey was only given to one medical school at one point in time. To better understand the barriers to mental health care in medical students, it would be better to compare multiple medical schools over multiple years and to follow students throughout their medical education.

## Conclusions

Mental health in medical providers is of paramount importance to achieving the highest level of patient care. Thus, we must elucidate the factors that lead to higher levels of anxiety and depression in the earliest stages of medical education. In this study, students at one medical school were surveyed to determine their levels of anxiety, stigma, and perceptions on barriers to mental health care. While high levels of students reported stigma as a barrier to care, students with higher levels of anxiety were more likely to report cost. Furthermore, preclinical students were more likely to report cost, despite access to free non-affiliated psychotherapy. We suggest that further work should be done to better understand how to relieve feelings of financial strain in the early years of medical school.

Clinical students in their third and fourth years were more likely to report stigma as a barrier to mental health care than preclinical students in their first and second years. We suggest this may be due to the impending residency applications, but also could be due to increased levels of mental health stigma that students experience while in their clinical rotations. The global pandemic of Covid-19 hit at different points along the medical school pathway for each class. It is possible that the pandemic effected the way students view mental health differently based on year.1 A longitudinal study should be done to follow medical students and identify when, and what experiences may change their perceptions to barriers of mental health care.

Finally, we recommend that each medical school curate a proactive mental health plan, which may include early individual sessions with a mental health provider, easily accessible mental health resources, and the promotion of open conversations regarding mental health struggles.

## Supplementary Information

**Additional file 1.**

## Data Availability

The datasets generated and/or analyzed during the current study are not publicly available due to student confidentiality but are available from the corresponding author on reasonable request.
