# Supplementary material for: Barriers to help-seeking in medical students with anxiety at the University of South Carolina School of Medicine Greenville
Source: BMC Med Educ. 2023 Jun 21;23:463. doi: 10.1186/s12909-023-04460-5 (PMC10286385; doi:10.1186/s12909-023-04460-5)
Supplement: Supplementary file 1 — Additional file 1. [file 12909_2023_4460_MOESM1_ESM.pdf]

Start of Block:

For the following questions chose the answer that most closely fits you. The only identifying information in this survey are your medical school class and gender. Age, and race demographics were left out to keep the data generated anonymous. If you begin the survey, you are free to stop taking the survey at any time.

Page Break

What medical school year are you in?

- ○ Year 1 (1)
- ○ Year 2 (2)
- ○ Year 3 (3)
- ○ Year 4 (4)

What is your gender identity?

- ○ Male (1)
- ○ Female (2)
- ○ Non-binary / third gender (3)
- ○ Prefer not to say (4)
- ○ Not listed (5) ____

End of Block

Start of Block: stigma

For the following questions mental health disorder refers to any mental health disorder (depression, anxiety disorders, schizophrenia, eating disorders and addictive behaviors, etc.)

Medical students with a mental health disorder could “snap out of it” if they wanted to do so.

- Strongly agree (1)
- Somewhat agree (2)
- Neither agree nor disagree (3)
- Somewhat disagree (4)
- Strongly disagree (5)

Most medical students would not want to work with a medical student with a mental health disorder.

- Strongly agree (1)
- Somewhat agree (2)
- Neither agree nor disagree (3)
- Somewhat disagree (4)
- Strongly disagree (5)

Medical students with a mental health disorder are not to blame for their problems

- ☐ Strongly agree (1)
- ☐ Somewhat agree (2)
- ☐ Neither agree nor disagree (3)
- ☐ Somewhat disagree (4)
- ☐ Strongly disagree (5)

Medical students with a mental health disorder are dangerous to their patients

- ○ Strongly agree (1)
- ○ Somewhat agree (2)
- ○ Neither agree nor disagree (3)
- ○ Somewhat disagree (4)
- ○ Strongly disagree (5)

Telling a counselor I was depressed would be risky to my career.

- ○ Strongly agree (1)
- ○ Somewhat agree (2)
- ○ Neither agree nor disagree (3)
- ○ Somewhat disagree (4)
- ○ Strongly disagree (5)

Other students would stop including me in social activities if they discovered I had a mental health disorder

- Strongly agree (1)
- Somewhat agree (2)
- Neither agree nor disagree (3)
- Somewhat disagree (4)
- Strongly disagree (5)

If I had a mental health disorder, I would seek treatment

- Strongly agree (1)
- Somewhat agree (2)
- Neither agree nor disagree (3)
- Somewhat disagree (4)
- Strongly disagree (5)

If I were depressed I would be blamed for being unable to cope

- Strongly agree (1)
- Somewhat agree (2)
- Neither agree nor disagree (3)
- Somewhat disagree (4)
- Strongly disagree (5)

Other students and faculty members would view me as unable to handle my responsibilities if I had depression or anxiety.

- ◯ Strongly agree (1)
- ◯ Somewhat agree (2)
- ◯ Neither agree nor disagree (3)
- ◯ Somewhat disagree (4)
- ◯ Strongly disagree (5)

If I were depressed fellow medical students would respect my opinions less.

- ☐ Strongly agree (1)
- ☐ Somewhat agree (2)
- ☐ Neither agree nor disagree (3)
- ☐ Somewhat disagree (4)
- ☐ Strongly disagree (5)

If I were depressed and applying to residency, my application would be less competitive to residencies than that of a student who does not have depression.

- ○ Strongly agree (1)
- ○ Somewhat agree (2)
- ○ Neither agree nor disagree (3)
- ○ Somewhat disagree (4)
- ○ Strongly disagree (5)

If I had anxiety and was applying to residency, my application would be less competitive to residencies than that of a student who does not have anxiety.

- ○ Strongly agree (1)
- ○ Somewhat agree (2)
- ○ Neither agree nor disagree (3)
- ○ Somewhat disagree (4)
- ○ Strongly disagree (5)

If I sought treatment for depression, my residency application would be less competitive to residencies than that of a student who did not seek treatment for depression.

- ○ Strongly agree (1)
- ○ Somewhat agree (2)
- ○ Neither agree nor disagree (3)
- ○ Somewhat disagree (4)
- ○ Strongly disagree (5)

If I sought treatment for anxiety, my residency application would be less competitive to residencies than that of a student who did not seek treatment for anxiety.

- Strongly agree (1)
- Somewhat agree (2)
- Neither agree nor disagree (3)
- Somewhat disagree (4)
- Strongly disagree (5)

If I were depressed, I would worry that my medical student friends who knew would tell other students or faculty.

- Strongly agree (1)
- Somewhat agree (2)
- Neither agree nor disagree (3)
- Somewhat disagree (4)
- Strongly disagree (5)

If I sought help for a mental health disorder I would worry that medical students and faculty would find out.

- ◯ Strongly agree (1)
- ◯ Somewhat agree (2)
- ◯ Neither agree nor disagree (3)
- ◯ Somewhat disagree (4)
- ◯ Strongly disagree (5)

A medical student who sees a counselor is admitting that he/she is unable to handle the stress of medical school

- ◯ Strongly agree (1)
- ◯ Somewhat agree (2)
- ◯ Neither agree nor disagree (3)
- ◯ Somewhat disagree (4)
- ◯ Strongly disagree (5)

Depression is a sign of personal weakness.

- ◯ Strongly agree (1)
- ◯ Somewhat agree (2)
- ◯ Neither agree nor disagree (3)
- ◯ Somewhat disagree (4)
- ◯ Strongly disagree (5)

Medical students with depression are not worth the time and resources for medical school teaching

- Strongly agree (1)
- Somewhat agree (2)
- Neither agree nor disagree (3)
- Somewhat disagree (4)
- Strongly disagree (5)

If I were depressed and asked for help, I would be admitting that my coping skills are inadequate.

- Strongly agree (1)
- Somewhat agree (2)
- Neither agree nor disagree (3)
- Somewhat disagree (4)
- Strongly disagree (5)

Medical students with depression are dangerous to their patients

- Strongly agree (1)
- Somewhat agree (2)
- Neither agree nor disagree (3)
- Somewhat disagree (4)
- Strongly disagree (5)

End of Block: stigma

Start of Block: Block 3

Over the last 2 weeks, how often have you been bothered by the following problems?

Feeling nervous, anxious or on edge.

- ○ Not at all (1)
- ○ Several days (2)
- ○ More than half the days (3)
- ○ Nearly every day (4)

Not being able to stop or control worrying.

- ○ Not at all (1)
- ○ Several days (2)
- ○ More then half the days (3)
- ○ Nearly every day (4)

Worrying too much about different things.

- ○ Not at all (1)
- ○ Several days (2)
- ○ More than half the days (3)
- ○ Nearly every day (4)

Trouble relaxing.

- Not at all (1)
- Several days (2)
- More than half the days (3)
- Nearly every day (4)

Being so restless that it is hard to sit still

- Not at all (1)
- Several days (2)
- More than half the days (3)
- Nearly every day (4)

Becoming easily annoyed or irritable

- Not at all (1)
- Several days (2)
- More than half the days (3)
- Nearly every day (4)

Feeling afraid as if something awful might happen

- ◯ Not at all (1)
- ◯ Several days (2)
- ◯ More than half the days (3)
- ◯ Nearly every day (4)

End of Block: Block 3

Start of Block: Block 5

If you were suffering from a mental health disorder, what would be the largest barrier to seeking mental health care for yourself?

- ◯ Write here: (1) ____

End of Block:
